# The role of boundary spanners in delivering collaborative care: a process evaluation

**DOI:** 10.1186/s12875-016-0501-4

**Published:** 2016-07-29

**Authors:** Carianne M. Hunt, Michael Spence, Anne McBride

**Affiliations:** 1 NIHR Collaboration for Leadership in Applied Health Research and Care (CLAHRC), Greater Manchester, Salford Royal NHS Foundation Trust, 3rd Floor Mayo Building, Stott Lane, Salford, M6 8HD Greater Manchester UK; 2 Manchester Business School, University of Manchester, Room E26, Booth Street East, Manchester, M15 6PB UK

**Keywords:** Boundary spanning, Co-ordinated care, Mixed methods, Severe Mental Illness (SMI), Primary care, Community care

## Abstract

**Background:**

On average, people with schizophrenia and psychosis die 13–30 years sooner than the general population (World Psychiatry 10 (1):52–77, 2011). Mental and physical health care is often provided by different organisations, different practitioners and in different settings which makes collaborative care difficult. Research is needed to understand and map the impact of new collaborative ways of working at the primary/secondary care interface (PloS One 7 (5); e36468). The evaluation presented in this paper was designed to explore the potential of a Community and Physical Health Co-ordinator role (CPHC) (CPHCs were previously Care Co-ordinators within the Community Mental Health Team, Community in the title CPHC refers to Community Mental Health) and Multi-Disciplinary Team (MDT) meetings across primary and community care, with the aim of improving collaboration of mental and physical health care for service users with Severe Mental Illness (SMI).

**Methods:**

Data collection took place across five general practices (GPs) and a Community Mental Health Team (CMHT) in the Northwest of England, as part of a process evaluation. Semi-structured interviews were conducted with a purposive sample of GP staff (*n*= 18) and CMHT staff (*n*=4), a focus group with CMHT staff (*n*=8) and a survey completed by 13 CMHT staff, alongside cardiovascular risk data and MDT actions. Framework analysis was used to manage and interpret data.

**Results:**

The results from the evaluation demonstrate that a CPHC role and MDT meetings are effective mechanisms for improving the collaboration and co-ordination of physical health care for SMI service users. The findings highlight the importance of embedding and supporting the CPHC role, with an emphasis on protected time and continuing professional roles and integrating multiple perspectives through MDT meetings. Considering the importance of physical health care for SMI service users and the complex environment, these are important findings for practitioners, researchers and policy makers in the field of primary care and mental health.

**Conclusion:**

There is an increasing focus on integration and collaborative working to ensure the delivery of quality care across the whole patient pathway, with a growing need for professionals to work together across service and professional boundaries. The introduction of a two pronged approach to collaboration has shown some important improvements in the management of physical health care for service users with SMI.

## Background

People with mental health problems, such as schizophrenia or bipolar disorder, die on average 13–30 years sooner than the general population [1, 2–4]. This mortality gap has widened in recent decades [5–7] and approximately 60 % is due to physical illness [8, 9]. Individuals with Severe Mental Illness (SMI) have increased frequency of tuberculosis, HIV, obesity related cancer, osteoporosis, stroke, hypertension, myocardial infarction, diabetes [1]. A greater predisposition to develop metabolic abnormalities in combination with the metabolic adverse effects of antipsychotic drug treatments can have a negative influence on physical health [10]. It is, however, possible to avoid many of these problems if close attention is paid to service users’ physical health [11].

Despite many of the physical health issues experienced by this group, (e.g. cardiovascular diseases and diabetes) being preventable or controllable by chronic disease management, people with SMI continue to experience health inequalities, particularly in relation to the provision of physical health care services. There is an enormous gap in physical health outcomes for those with mental health problems and, as such, more needs to be done to promote and manage good mental health and prevent mental ill health [12]. These inequalities are attributed to a combination of factors, one of which being the separation of mental health services from other medical services [13, 14] and poor clarity regarding responsibility in care co-ordination [15]. When service users do access health services, their physical health needs are often ignored or treated as a manifestation of their mental health condition, rather than a health issue. This ‘diagnostic overshadowing’ [16] can lead to physical conditions being undiagnosed and untreated, which can prove fatal. Aligning health services to improve the co-ordination and collaboration of care could lead to improvements for those in greatest need. The increased prevalence of cardiovascular disease and its modifiable risk factors highlights the importance of ensuring that people with SMI are monitored and screened regularly.

Terms such as ‘collaborative care’, ‘shared care’ and ‘integrated care’ are often used interchangeably, however, a common theme running through all definitions is a focus on developing closer working relationships between primary care (family doctors or GPs and practice nurses) and specialist health care (such as Community Mental Health Teams). Collaboration between mental health care providers and GPs is important, particularly as many patients with SMI are treated as outpatients. Collaboration should be organised around systematic and structural collaboration between GP and mental health care services, including clearly defined responsibilities, with a focus on a patient centered, holistic and tailored approach to caring for patients with SMI [17]. Further research in this area is needed to improve the co-ordination of care for patients with complex needs and long-term illness, which is currently described as poor [18, 19]. It is important for the health and wellbeing of service users with mental and physical multimorbidity that future research explores how integrated care models can be translated into routine primary care [20].

Physical health care of SMI service users does not occur as a single or isolated event, rather as a complex series of linked incremental activities which can involve a diverse range of occupational groups and span a range of organisational boundaries. The evaluation presented in this paper accepts the complexity of this area and focuses on understanding *how* a boundary spanning role can assist healthcare professionals in improving the collaboration of physical health care for SMI service users. Boundary spanners help to *“facilitate transactions and the flow of information between people or groups who either have no physical or cognitive access to one another, or alternatively, who have no basis on which to trust each other”* [21]. Despite the emergence of the concept of ‘boundary spanners’ and the potential of this role, the processes by which they can make improvements in knowledge sharing and integration in healthcare are yet to be determined. There is limited empirical research exploring the function of such individuals in healthcare systems, particularly examining how boundary spanners perform (or should perform) their role to improve quality of care [22]. The findings presented in this paper describe how a CPHC role and MDT meetings can work across the boundaries of primary and community care, facilitating the sharing and integration of information within and across services.

## Methods

The aim of the evaluation presented in this paper was to understand the function of the CPHC role as a boundary spanner and how the role was operationalised alongside MDT meetings, with the aim of combining and integrating multiple perspectives.

The research was conducted by the Collaboration for Leadership in Applied Health Research and Care, Greater Manchester (CLAHRC GM) between June 2012 and December 2013. Two members of staff from the CMHT were seconded on a part time basis to the project as CPHCs, both secondees worked part time as Care Co-ordinators (CCs) within the CMHT. The intervention described in this paper was designed to implement a CPHC role and to co-ordinate MDT meetings between primary and community care to improve the physical health management of people with SMI, for example, managing lifestyle issues and disease reviews.

A purposive sample of key informants was chosen because of their involvement in the programme and to represent a range of views based on the care of SMI service users. Participants were interviewed from all participating GP practices and included medical, nursing and administrative staff.

The authors confirm that formal ethical approval was not required for the study since it was classified by National Health Service (NHS) governance procedures as service evaluation. All participants received a participant information sheet and consented to the recording of their interview and use of their data.

### Data collection

Eighteen semi-structured interviews were conducted with a range of medical, nursing and administrative staff across primary care and four semi-structured interviews with staff from the CMHT. A focus group was conducted with CCs (*n*=8) and a survey administered with CCs (*n*=13) (see Table 1).

**Table 1:** Data collection

Data collection	Participants	Number
Interviews	GP staff	18
Interviews	CPHCs	2
Interviews	CMHT Managers	2
Focus group	CMHT staff	8
Questionnaire	CMHT staff	13

Prompt sheets were prepared prior to the semi-structured interviews to guide data collection and included the following themes: relationship with CMHT and primary care, the role of the CMHT and primary care teams, knowledge of physical health care for SMI service users, the role of the CPHC, collaborative and integrated care across the CMHT and primary care. The prompt sheets were used to guide and focus discussion, however the interviews were flexible and participants were able to discuss any relevant issues. The semi-structured interviews enabled participants to reflect on their individual roles and provide key information in relation to the co-ordinating role and the MDT meetings and specifically the process of facilitating collaborative care. The focus groups were an important source of information as they provided an opportunity for CMHT staff to discuss themes from the perspective of community care and from their individual experiences of working with service users in the community. All interviews were conducted at the participants’ place of work, GP surgery, CMHT team offices, or over the telephone, whichever was most convenient. All interviews were audio-recorded and transcribed verbatim.

Alongside the interviews, quantitative data was collected to show the number and type of MDT actions performed and completed (see Fig. 3) and anonymised cardiovascular risk data (based on the indicators required for QRISK2^1^) for each service user under the care of the CMHT and the pilot practices (see Table 2). This data focussed on the QRISK2 key measurement criteria, i.e. weight/body mass index (BMI), blood pressure, cholesterol and recorded smoking status, for the previous 12 months. Baseline data was collected between June and July 2012 and a re-audit at 9 months (March and April 2013).

**Table 2:** Cardiovascular risk data

	Service users	Practices
Baseline	186	5
Re audit	172^5^	5

^5^Numbers differ due to natural flux in the case load of the CMHTs. All results were adjusted accordingly

### Framework analysis

A coding scheme was designed based on the topic areas previously discussed and data was analysed using an analytic hierarchy approach. The stages of this approach included identifying themes and concepts related to the co-ordination of care and the implementation of the CPHC role. Data was then labelled by themes, summarised and refined, any new categories were also identified. Explanations of the data were then developed, looking specifically at how and why [23]. As interview transcripts were produced they were read by the authors who made notes of emerging themes and concepts, these were then labelled and sorted by theme. Categories were then identified and any patterns were recorded alongside explanations of the data.

## Results

Analysis identified three main components of the CPHC role and MDT meetings, the importance of embedding and supporting the role in practice and facilitating the integration of multiple perspectives, below we elaborate on each of these areas.

### The CPHC an embedded boundary spanner

Prior to the CPHC role communication between primary and community care was *‘patchy’*, *‘sporadic’* and *‘disjointed’,* which led to limited co-ordination across services. Individual electronic patient records are held by multiple organizations, however there is no interoperability between the systems, and the sharing of patient related information is limited. The current mechanism involves the sharing of a paper based Care Planning Approach (CPA) document between the services; however the physical health information held within the CPA is often limited, insufficient and outdated.

*“I think historically, there could have been a bit of tension, GPs wanted us to do things, we wanted GPs to do it and it would end up nobody doing anything. I think in most surgeries we work with, that has gone. So that’s really good.”* (CPHC, CMHT)

The CPHC acted as a co-ordinator across primary and community care, spanning service boundaries, with a focus on improving communication and collaboration. The role was pivotal for facilitating information sharing between the GP practices and CMHT, which helped to improve working relationships between services and promoted a greater understanding and respect for professional roles. CPHCs provided a key link between primary and community care which enabled CCs to provide a more holistic care plan for service users. CPHCs were seen as a channel or conduit for transferring and integrating service user knowledge, which helped to establish a clear path for maintaining communication and sharing information, as the following quotes illustrate.

*“Yes I do think care is more co-ordinated, just through attending the meetings, we’re now aware what patients are being seen, what care is being given to those patients, and we’re more aware of those patients ourselves.” (*Practice Manager, Primary Care)

*“Liaison with the [community physical health co-ordinator] is time saving for Care Co-ordinators and enables a better package of care for the client.”* (Care Co-ordinator, NW CMHT)

*“I think the GPs understand who people are and I think they understand the roles of people better. I think there was almost a light bulb switched on in one meeting when they realised the breadth of the CMHT, and also the individuals who look after it.” (*Practice Manager, Primary Care)

The two CPHCs were assigned to the position for 0.4 Whole Time Equivalent (WTE) (each), the remainder of the time they continued in their CC role. This split function was crucial for embedding the CPHC role in practice as it allowed CPHCs to retain their CC skills, retain access to appropriate service user and organisational information, meetings and discussions and importantly helped to maintain trust and respect from peers.

*“Because there’s the side to it that you’re still embedded in the team, and still carry a care coordination case load, which as an individual, they’ve said, keeps their skills at the right level, but it also lends a credibility to their role in the team, because they are a part of that team.”* (CMHT Manager)

*“They (CPHCs) have the knowledge and contact they’ve had with that patient, and they can explain clearly about what the patient is going through, what the patient needs, and any actions can be agreed pretty much immediately”* (Practice Manager, Primary Care)

Primary and community care staff suggested that continuing to be embedded in the team was an important aspect of the role for maintaining trust and accessing relevant service user information, both of which were crucial to the successful implementation of the role.

### Supporting boundary spanners

Management support and active engagement were seen as critical elements to success in terms of understanding the CPHC role and providing support, guidance and supervision. It was imperative for CMHT management to have an understanding of the commitment required to perform the role and the ability to make necessary changes to individual caseloads. Indeed, the importance of protected time for this role was noted by one of the CPHCs, a colleague and two managers:

*“It is key to the CPHC as a separate, dedicated role, and to give the role protected time and reduced case load.”* (CPHC, CMHT)

*“Yeah, I think it’s a full-time job but I think like […] and […] have probably struggled to do it around other commitments and I think if it’s a permanent role I think that should just be their job.”* (CC, CMHT)

*“it would be very important to have that [CPHC role] as a distinct role because again, bolting that onto a care co-ordinator without the allowance of the amount of time that kind of role would take would be…again it would be one of those things that gets lost as a priority in terms of that liaison with the GP practices and for the general medical sort of side.”* (CMHT Manager)

*“I think people would struggle to do it as a tag on to the care coordinator …they [CPHCs] have that dedicated time to go to the GP practices, and to do all the follow up around that. So they’re not carrying a full case load and then having to do it on top of it, because with prioritisation, the link worker role would naturally go to one side if you carry a full case load, because we have that many competing priorities, and ultimately a lot of the priorities is around our performance”* (CMHT Manager)*.*

Commitment to the role was important for providing the CPHCs with an official mandate to perform their responsibilities, which also added legitimacy to the role, the importance of this should not be underestimated.

Evaluation findings suggest that providing protected time for the role enabled the CPHCs to develop systems to improve the flow of communication and information within and between services. CPHCs were self-organising in that they worked with and across teams to design ways of working that were tailored to the local setting, for example, designing MDT meetings based on the GP setting (i.e. size of practice), small practices held small MDT meetings with GPs. Community and primary care staff worked together to develop a flowchart of responsibilities for the CPHC role which helped to provide some consistency and continuity across different teams. This helped to ensure that processes were tailored to the individual setting, rather than taking a general approach to implementation. CPHCs designed and developed a method for identifying service users for discussion at the MDT meetings. This identification process was accompanied by a method of tracking actions from the MDT meetings, ensuring that the CPHC, CC and primary care staff were fully aware and informed about what had been discussed and agreed at the meeting and what outcomes required actions.

*“The feedback from the surgeries is fantastic. They really like the form that we’ve come up with in the last month or two, with the colour coding about whether actions have been completed or not, with all the clients that we’ve discussed.”* (CPHC, CMHT)

#### Integrating multiple perspectives around the service user

**Fig. 1:**
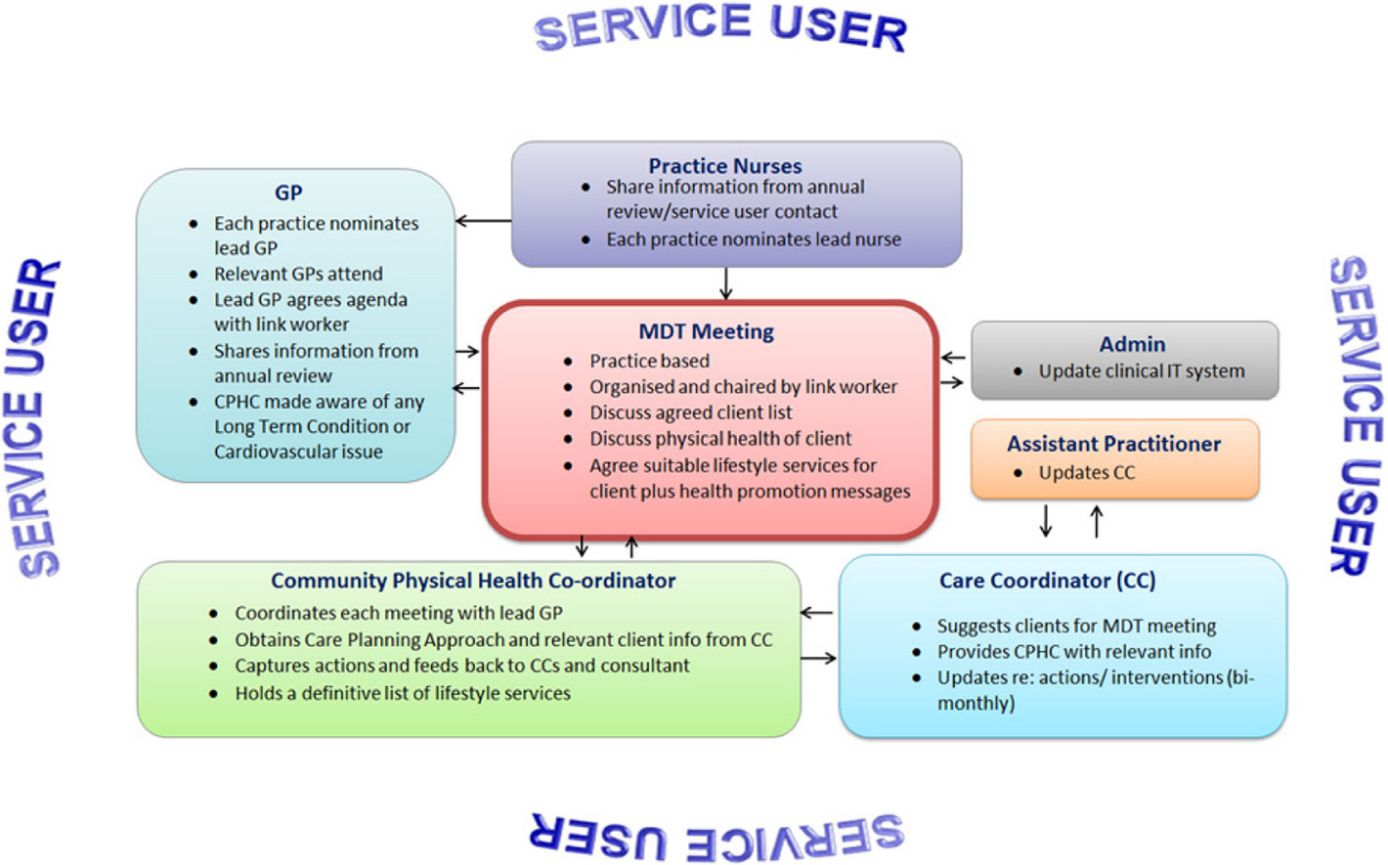
The MDT meeting

While the CPHC was seen as a conduit for information sharing and communication, the MDT meetings were designed as a vehicle for processing this information, with an aim of integrating and building effective action plans for service users. MDT meetings were conducted in a variety of ways across the teams (see Fig. 1). The general consensus, however, was to involve, at a minimum, a GP, practice manager/administrator, practice nurse/health care assistant and CPHC and to hold the meetings monthly or bi monthly. The importance of local requirements and delivery were taken into account, with some MDT meetings designed as a standalone meeting and others forming part of a wider integrated care meeting, for example, integrated into existing palliative care or long term conditions meetings. Figure 1 provides an illustration of the various aspects of the care model described in this article, highlighting the key method of collaboration, the MDT meeting and the various health care professionals who are required to input into these meetings and the various information that they are required to provide.

Gathering multiple perspectives and sharing information across services enabled the teams to provide a co-ordinated approach to the care of service users. MDT meetings were designed to be a mechanism to share information with an action orientated focus, with all meetings producing some form of action plan for each service user discussed. These actions included referring service users to lifestyle programme, medication reviews, disease reviews, for example repeat blood investigations, diabetes checks (see Fig. 3). Attendees at the MDT meetings designed an action plan for the service users based on their integrated knowledge; this action plan was then communicated to relevant professionals across and within services.

Actions plans were then discussed and reviewed at MDT meetings. The processes, which were designed to help facilitate the flow of communication, were achieved through the CPHCs working with CMHT staff and primary care to design local solutions to meet local requirements. For example, one GP practice found that the MDT action plans were not used effectively, to address this issue the team used the plan-do-study-act (PDSA)^2^ cycle to test new ways of working. The process was adapted to include a colour coded MDT traffic light system as a way to clearly decipher the responsibility for actions arising from the MDT meeting. These codes were used alongside scheduled phone calls with the practice manager to provide progress updates between MDT meetings. CPHCs shared information from the MDT meetings through the traffic light form, or similar documentation, which provided an overview of the actions discussed during the MDT meetings and the individual(s) responsible. The traffic light system was used to denote the status of action: actions are complete (green), action performed but needs following up (amber), action needs to be performed or the existing action is outstanding (red). CPHCs produced a clinical guidance document to assist future CPHCs, particularly in relation to the process of preparing, attending and following up actions from MDT meetings.

**Fig. 2:**
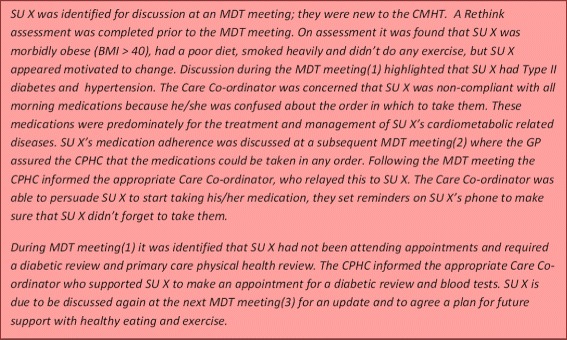
Service user case study – CPHC perspective

Engagement with MDT meetings was important for developing an improved understanding of service user’s physical health care needs which was a key facilitator for designing and delivering appropriate physical health care management plans. The MDT meetings provided primary care and community staff with a space to share and acquire knowledge concerning service users’ physical and mental health in a supportive environment. The meetings were an important opportunity for staff to reflect on the care of service users and update other key professionals, identifying the availability of, and gaps in knowledge. The primary care team were able to provide detailed information about service users’ physical health, for example, the severity of their physical condition and any recent assessments or screening and the CPHCs provided information on other factors impacting physical health, such as medication adherence and lifestyle. This collaboration and co-ordination of knowledge facilitates a holistic approach to care, which is essential for SMI service users as their physical health may be deteriorating because of a lifestyle issue which may be exacerbated by their mental health. The MDT meetings provided an opportunity for staff to understand the relationship between mental and physical health; understanding how physical health impacts on mental health and vice versa. Knowledge integration was focused on combining knowledge about service users from multiple perspectives around a key objective, rather than simply making information and data available. Figure 2 and the following quotes illustrate how knowledge was combined in the MDT meetings to improve the provision of care.

*“The service user had raised cholesterol, I identified that the literature that had been provided to the client regarding healthy eating had not helped. Following discussion in the MDT it was agreed that he would be appropriate for one to one healthy eating advice from the practice nurse. Appointments made and client attended.” (*Care Co-ordinator, CMHT)

*“They [CPHCs] have the knowledge and contact they’ve had with that patient, and they can explain clearly about what the patient is going through, what the patient needs, and any actions can be agreed pretty much immediately”* (Practice Manager, Primary Care)

*“Increased knowledge about the client: I think you find out things about service users that you and the care co-ordinator and the consultant weren’t aware of and* vice versa*.”* (CPHC)

**Fig. 3:**
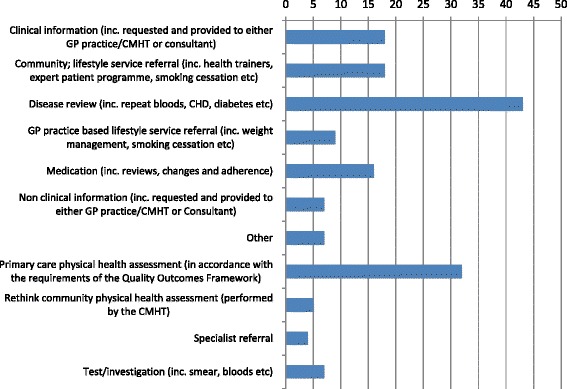
MDT actions

The MDT meetings provided a forum to discuss an array of physical health care needs, Fig. 3 shows the breadth and frequency of issues discussed during MDT meetings. The figure illustrates the level of integration across MDT meetings, highlighting the range of different actions, from improving the recording of clinical information and following up with lifestyle service referrals to ensuring that CCs are engaged in the process of helping to engage service users. Engagement is particularly important for ensuring service users have critical disease and medication reviews and attend for crucial tests or investigations. These MDT actions show how CPHCs were able to act as a key conduit of information and a crucial point of engagement across primary and secondary care, ensuring that these important actions were followed up and reviewed. Disease reviews (*n*=43; 26 %) were the most frequently discussed issue, closely followed by actions relating to physical health checks in both primary care (*n*=32; 20 %) and in the community (via Rethink community physical health assessment (CPHA) *n*=5; 3 %). Medication reviews, changes and discussions around adherence levels were also prominent in MDT meetings (*n*=15; 9 %). These findings demonstrate the focus of MDT meetings and the importance of sharing information across services. To understand how MDT actions were performed and the process involved, we collected accountability data which showed who was responsible for each MDT action. Encouragingly, we found an equal division of responsibility for actions (GP *n*=34; 21 %, Practice Nurse *n*=19; 12 % and combined *n*=53; 33 %) and the CMHT (CC *n*=58; 36 %), with a number of these actions requiring joint responsibility (*n*=47; 29 %). This shows that primary and community staff worked together to implement MDT actions, which demonstrates a collaborative approach to physical health care management and suggests that collaborative care was extended beyond the MDT meetings.

Alongside the qualitative data, GP practice clinical systems were re-audited to investigate any changes to reported cardiovascular risk clinical information (using QRISK2 indicators). The data showed that there was a marked improvement of 25.7 % in the quantity of information recorded. During the start-up of the project there was ‘data rich’ QRISK2 scores (‘data rich’ = QRISK2 scores in which the following four factors were always recorded: systolic blood pressure, HDL: cholesterol ratio, smoking status and body mass Index) for 38 % of service users (*n*=115), post project this had improved to 58 % (*n*=79).^3^ At the point of data collection not all of the service users had been discussed at an MDT meeting. As the meetings continue we expect that the number of service users discussed will increase and in turn there will be an increase in the follow up QRISK2 data. Service users were required to attend the GP practice for the GP and/or nurse to perform the relevant QRISK2 assessments, completing this information helped to establish a clearer picture of service users’ physical health.

Despite improvements in knowledge sharing, respondents commented on the continued problem of accessing hard to reach groups. Suggesting that the role of the CPHC had helped to improve care for some service users, however, further work was needed to access more difficult to reach groups, arguably those in more need of a co-ordinated approach.

*“I think we probably have made contact with some patients, who we wouldn’t ordinarily have seen. But there are still the hard-core that we don’t get through to”* (GP, primary care)

## Discussion

Individuals who occupy boundary spanning roles facilitate the communication and sharing of expertise, linking groups who might be separated in terms of location, division, or function [24]. There are generally two types of boundary spanner, the first are those who have a dedicated role to work as a boundary spanner across organisations or settings and connect different groups [25] and the second are those individuals who undertake boundary spanning activities as part of their mainstream job. The CPHCs described in this paper were embedded in the community care team with dedicated time to perform the role. The key function of their role was to act as a bridge between professionals and services, spanning the boundaries of primary and community care. Boundary spanning activities focused on improving the sharing and co-ordination of communication, with an emphasis on mobilising a shared willingness to collaborate. In essence, the role was designed to facilitate knowledge transfer and integration, allowing each group of professionals to present their specific knowledge in a structure and format that could be integrated with individuals on either side of the boundary. CPHCs were able to transfer knowledge from primary and community care and apply this to each service user, which provided an opportunity for emergent and shared meaning [25]. The CPHC role was developed through “learning by meeting”, as attendance at MDT meetings was a crucial opportunity for building personal and collective practice [26, 27].

Protected time was a key factor for success and enabled the CPHCs to be self-organising. They were responsible for liaising with teams from primary and community care to design a method of working which was appropriate, relevant and locally tailored to the needs of the service. By appointing an individual to this role, rather than simply implementing a set of guidelines and protocols, a process was developed whereby changes and improvements were designed and embedded into practice. An example of this self-organisation was the design of the MDT meetings, which was based on the GP setting (i.e. size of practice), small practices held small MDT meetings with GPs, practice managers, CPHCs and occasionally practice nurses, larger practices held more traditional *‘integrated care’* meetings including a wide variety of healthcare professionals, such as specialist nurses and teams (see Fig. 1). The CPHCs were self-organising, adapting their processes in line with the local needs and requirements of the service, relying on existing interdependencies, i.e. the relationships and interconnections between staff. The CPHC facilitated the MDT meetings by leveraging existing relationships and forging new relationships between primary and community care. CPHCs maintained their CC role which was critical for developing credibility, a finding supported by the literature which highlights the importance of boundary spanners acting as legitimate peripheral participants in the communities with which they are working and maintaining a strong professional identification [28, 29]. As Wenger (1998: 109) states, the role *“requires enough legitimacy to influence the development of a practice, mobilize attention, and address conflicting interests. It also requires the ability to link practices by facilitating transactions between them, and to cause learning by introducing into a practice elements of another.”* [25].

While the CPHC was seen as a boundary spanner and conduit for sharing communication, the MDT meetings were viewed as a mechanism by which multiple perspectives could be shared and integrated to develop and build effective management plans for service users. Knowledge integration was focused on combining service user data and information from multiple perspectives around key objectives with an action orientated focus, rather than simply sharing information in a passive way. As this was a new process the team were able to co-evolve and develop new ways of working and new processes to communicate, share and integrate knowledge to improve the physical health care of service users. The role of the CPHC and MDT meetings were not simply to provide a ‘mechanistic’ passing and processing of information from one service to the other, but to find a way to develop what Carlile [30] describes as a three stage process of representation, learning and transformation. Health care staff had direct contact with the MDT meetings (through attendance), or by indirect contact (providing updates and information to the CPHC). The information gathered prior to the MDT meetings was used to build layers of understanding of service users’ physical health care needs [12]. The information was then discussed exploring different perspectives and developing a holistic picture of the service user and their care needs. This sharing and developing of knowledge was a critical part of the process which facilitated transformation, where existing knowledge was refined and individuals worked together to collaboratively develop ‘new’ knowledge. The process of representation, learning and transformation, which concluded in the creation of ‘new’ service user knowledge, helped to tailor engagement and care to the service users’ needs. CPHCs facilitated the process of integrating knowledge by coordinating service user information, personal know-how and experience of individuals, with a focus on problem-solving [31]. Integrating diverse knowledge from multiple sources, often across organisational boundaries, is challenging, but imperative for delivering collaborative care. The MDT meetings provided primary and community care staff with a space to discuss the availability of information and highlight significant gaps in knowledge.

### Strengths and weakness of the research

Considering the complexity of the environment and the importance of collaboration and co-ordination of care, this research is an important addition to the extant literature. The research focused on understanding *how* physical health care of SMI service users could be improved, rather than focusing on specific physical health outcomes, such as BMI. The research emphasises the importance of recognizing *“that we are dealing with issues of complexity and that there is no ‘one-size-fits-all’ solution to the challenge of knowledge translation in healthcare”* [32], by illustrating how a co-ordinating role can be used to facilitate the process of communication and co-ordination across service boundaries. Although the study was focused on one region of England, the findings can be generalised to other primary and community health care settings, however it is important to understand self-organisation within each setting to appreciate the variation across local contexts [33]. The findings presented in this paper provide some of this context by showing how the CPHC work with primary and community care to facilitate the process of collaboration. Rigour was addressed by collecting data from a variety of healthcare professionals across primary and community care and adopting a mixed method approach to data collection, to extend understanding and add depth and breadth to the analysis [23]. Data analysis was conducted by more than one person in the project team and the framework analysis method ensured that there was consistency in the data collection and analysis process.

The data clearly indicates improvements made in knowledge sharing and service user care management, however respondents commented on the continued problem of accessing hard to reach groups. Therefore, further work is needed to explore access issues for more difficult to reach groups.

The CMHTs were provided with funding for the two CPHCs (0.4 WTE each), therefore their views may not be typical of other CMHT staff in practices that did not take part in this work.

## Conclusions

Data indicates that the introduction of a two pronged approach, through a CPHC role and MDT meetings, has improved the management of physical health care for people with SMI, particularly through sharing of information, co-ordination of actions, and proactive delivery of care. The findings suggest that a a co-ordinating role, alongside MDT meetings can help to improve communication and collaboration across primary and community care. Collaboration of care for SMI service users has led to the design of joint action plans for the physical health management of service users which can now be discussed and appraised at MDT meetings. This is an important finding considering the gap in physical health outcomes and evidence that suggests co-ordinated and integrated physical care of patients with SMI has the greatest chance of improving physical health care outcomes [34].

## Abbreviations

CC, Care Co-ordinator; CLAHRC GM, Collaboration for Leadership and Applied Health Research and Care Greater Manchester; CMHT, Community Mental Health Team; CPHA, Community Physical Health Assessment; CPHC, Community and Physical Health Co-ordinator; GP, General Practitioner; MDT, Multi-Disciplinary Team; NHS; National Health Service; NIHR, National Institute of Health Research; NWCMHT, North-West Community Mental Health Team (NWCMHT); PCT, Primary Care Trust; SMI, Severe Mental Illness; WTE, Whole Time Equivalent

## Footnotes

1 QRISK cardiovascular disease risk algorithm (QRISK2) provides an accurate estimate of cardiovascular risk in patients from different ethnic groups in England and Wales. It relies on the following clinical indicators:a) age, b) gender, c) ethnicity, d) smoking status, e) diabetes status, f) family history of angina or myocardial infarction, g) chronic kidney disease, h) atrial fibrillation, i) blood pressure treatment, j) rheumatoid arthritis, k) cholesterol/HDL ratio, l) systolic blood pressure, and m) body mass index

2 The Plan Do Study Act (PDSA) cycle is part of the Institute for Healthcare Improvement Model for Improvement. It is a simple tool for accelerating quality improvement www.ihi.org

3 Numbers of service users differ from baseline to audit due to natural flux in the case load of the CMHTs. All results were adjusted accordingly.
